# Identification and application of a growth-regulated promoter for improving l-valine production in *Corynebacterium glutamicum*

**DOI:** 10.1186/s12934-018-1031-7

**Published:** 2018-11-24

**Authors:** Yuechao Ma, Yi Cui, Lihong Du, Xiaoqian Liu, Xixian Xie, Ning Chen

**Affiliations:** 1 National and Local United Engineering Lab of Metabolic Control Fermentation Technology, Tianjin University of Science & Technology, Tianjin, 300457 People’s Republic of China; 2 College of Biotechnology, Tianjin University of Science & Technology, No. 29, 13 Main Street, Tianjin Economic and Technological Development Area, Tianjin, 300457 People’s Republic of China

**Keywords:** *Corynebacterium glutamicum*, Growth-regulated promoter, Transcriptional regulation, Pyruvate dehydrogenase, Citrate synthase, l-Valine

## Abstract

**Background:**

Promoters are commonly used to regulate the expression of specific target genes or operons. Although a series of promoters have been developed in *Corynebacterium glutamicum*, more precise and unique expression patterns are needed that the current selection of promoters cannot produce. RNA-Seq technology is a powerful tool for helping us to screen out promoters with expected transcriptional strengths.

**Results:**

The promoter P_CP_2836_ of an aldehyde dehydrogenase coding gene from *Corynebacterium glutamicum* CP was identified via RNA-seq and RT-PCR as a growth-regulated promoter. Comparing with the strong constitutive promoter P_tuf_, the transcriptional strength of P_CP_2836_ showed a significant decrease that from about 75 to 8% in the stationary phase. By replacing the native promoters of the *aceE* and *gltA* genes with P_CP_2836_ in the *C. glutamicum* ATCC 13032-derived l-valine-producing strain AN02, the relative transcriptional levels of the *aceE* and *gltA* genes decreased from 1.2 and 1.1 to 0.35 and 0.3, and the activity of their translation products decreased to 43% and 35%, respectively. After 28 h flask fermentation, the final cell density of the obtained strains, GRaceE and GRgltA, exhibited a 7–10% decrease. However, l-valine production increased by 23.9% and 27.3%, and the yield of substrate to product increased 43.8% and 62.5%, respectively. In addition, in the stationary phase, the intracellular citrate levels in GRaceE and GRgltA decreased to 27.0% and 33.6% of AN02, and their intracellular oxaloacetate levels increased to 2.7 and 3.0 times that of AN02, respectively.

**Conclusions:**

The P_CP_2836_ promoter displayed a significant difference on its transcriptional strength in different cell growth phases. With using P_CP_2836_ to replace the native promoters of aceE and gltA genes, both the transcriptional levels of the *aceE* and *gltA* genes and the activity of their translation products demonstrated a significant decrease in the stationary phase. Thus, the availability of pyruvate was significantly increased for the synthesis of l-valine without any apparent irreversible negative impacts on cell growth. Use of this promoter can enhance the selectivity and control of gene expression and could serve as a useful research tool for metabolic engineering. 
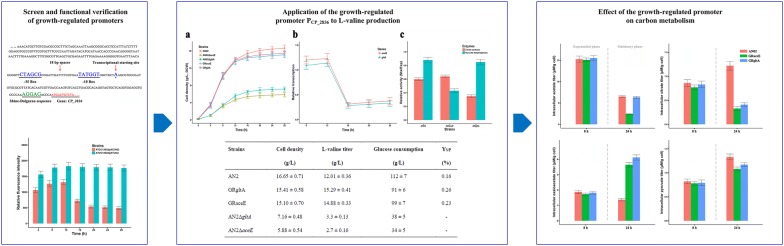

**Electronic supplementary material:**

The online version of this article (10.1186/s12934-018-1031-7) contains supplementary material, which is available to authorized users.

## Background

Promoters control the transcription of genes in bacteria and are commonly used to regulate the expression of target genes or operons in research involving metabolic engineering [1]. A series of promoters have been developed for use in protein expression in *Corynebacterium glutamicum* [2]. Strong constitutive promoters, such as the *tuf* and *sod* promoters, are widely used to optimize metabolic flux for the purpose of improving productivity and yield of target metabolites [3, 4]. This type of promoters enable constitutive expression of the target gene and are not affected by transcriptional regulators [5]. Thus, a target gene under the control of such promoters can be steadily expressed at a high level during all growth phases. However, in some cases such as the expression of toxic proteins, a constitutive expression is not suitable, where a tightly regulated and tunable promoter system is needed [6, 7]. An inducible promoter can deliver high levels of gene expression during a specific growth phase, as desired, and typically confers low expression levels prior to being activated [8]. Additionally, transcriptional repression and RNA interference can also be used to regulate the expression of target genes [9, 10]. It is performed by modifying the upstream DNA sequence or mRNA to inhibit the transcription or translation activity, subsequently weakening the expression of the gene. Moreover, CRISPR interference technology can be employed to specifically reduce the expression of target genes in *C. glutamicum* [11]. Together, these approaches can easily achieve regulated gene transcription by the simple addition of an inducer or a change to the culture conditions. However, during industrial scale fermentation, a small change in conditions can lead to significant fluctuations in production. Moreover, some specific requirements for controlled gene expression need highly precise and unique controls that current gene expression regulation methods cannot meet.

Under aerobic conditions, the TCA cycle is the main source of cellular energy and also provides a large number of organic acids as metabolic precursors [12, 13]. However, during the synthesis of some products, such as l-valine and l-leucine, an active TCA cycle results in unused carbon, which is not conducive to the efficient synthesis of target products. As such, there is a trade-off between product synthesis and the TCA cycle. Inactivation of the pyruvate dehydrogenase complex (PDHC) by deletion of the *aceE* gene can prevent pyruvate from converting to acetyl-CoA and further entering into the TCA cycle, which facilitates the synthesis of l-valine [14, 15]. Similarly, the inactivation of citrate synthase by deletion of the *gltA* gene can also weaken the TCA cycle while improving the efficiency of l-leucine synthesis [16]. However, although such genetic modifications improved the availability of carbon for the synthesis of target products, they also have an irreversible negative impact on cell growth, leading to a decline in the final overall production. It would therefore be desirable to find a way that could optimize carbon availability without a negative impact on cell growth for the transcription of those genes.

Transcriptomic analysis can give us a clear understanding of gene transcriptional patterns [17]. In our previously study, a strong constitutive promoter P_CP_2454_, was identified via RNA-seq from an l-leucine-producing mutant of *C. glutamicum* CP. The transcriptional strength of the P_CP_2454_ promoter was about 80% that of the *tuf* and *sod* promoters. This promoter could be used to strengthen gene expression and increased the production of l-valine in *C. glutamicum* [18]. In this study, statistical analysis of the RNA-seq data of *C. glutamicum* CP was used to identify genes with significant differences in transcription levels between the exponential and stationary phases. Genes that met this criterion might be controlled via a growth-regulated promoter, whose transcriptional strength was down-regulated when cells approach stationary phase [19]. The transcriptional strength of one such promoter, P_CP_2836_, was verified experimentally, and the promoter was applied to the construction of l-valine-producing strains to increase carbon availability and l-valine production. In addition, this growth-regulated promoter could be employed for engineering optimal gene expression in other *C. glutamicum* strains.

## Results and discussion

### Identification and functional verification of a growth-regulated promoter

The RNA-seq data of the mutant l-leucine-producing strain *C. glutamicum* CP were normalized and presented as the FPKM (expected number of Fragments Per Kilobase of transcript sequence per Million base pairs sequenced) [20]. Transcriptional levels of all genes between the cells in the exponential and stationary growth phases were compared using the average value of the FPKM of each sample (three independent repeats per sample). We first filtered out those genes for which the sum of FPKM values in the exponential growth phase and the stationary growth phase was < 100 to ensure that the transcriptional strength of the screened promoters had a considerable value. As a result, a total of 1836 genes remained. After a log10-fold change, the absolute value of FPKM ratios for about 90% of the genes was < 0.36, which was considered an insignificant differential expressions between the exponential and stationary growth phases. The FPKM ratio of the *tuf* gene was 0.02, which was under the control of the frequently used strong constitutive *tuf* promoter and is considered as very stable. The highest FPKM ratio (1.62) was demonstrated by *CP_2836*, an aldehyde dehydrogenase coding gene, which indicated that the transcriptional level of *CP_2836* in the exponential growth phase was significantly higher than that in the stationary growth phase.

To verify this observation, the transcriptional level of *CP_2836* was measured by fluorescence quantitative RT-PCR in reference to *tuf*. In the exponential growth phase, the transcriptional level of *CP_2836* was about 75% that of *tuf*. In the stationary growth phase, however, the level of *CP_2836* decreased to just about 8% that of *tuf*. This result indicated that the transcription of *CP_2836* was controlled by a growth-regulated promoter, whose transcriptional strength was significantly decreased in the stationary growth phase.

### GFP expression under control of the growth-regulated promoter

**Fig. 1:**
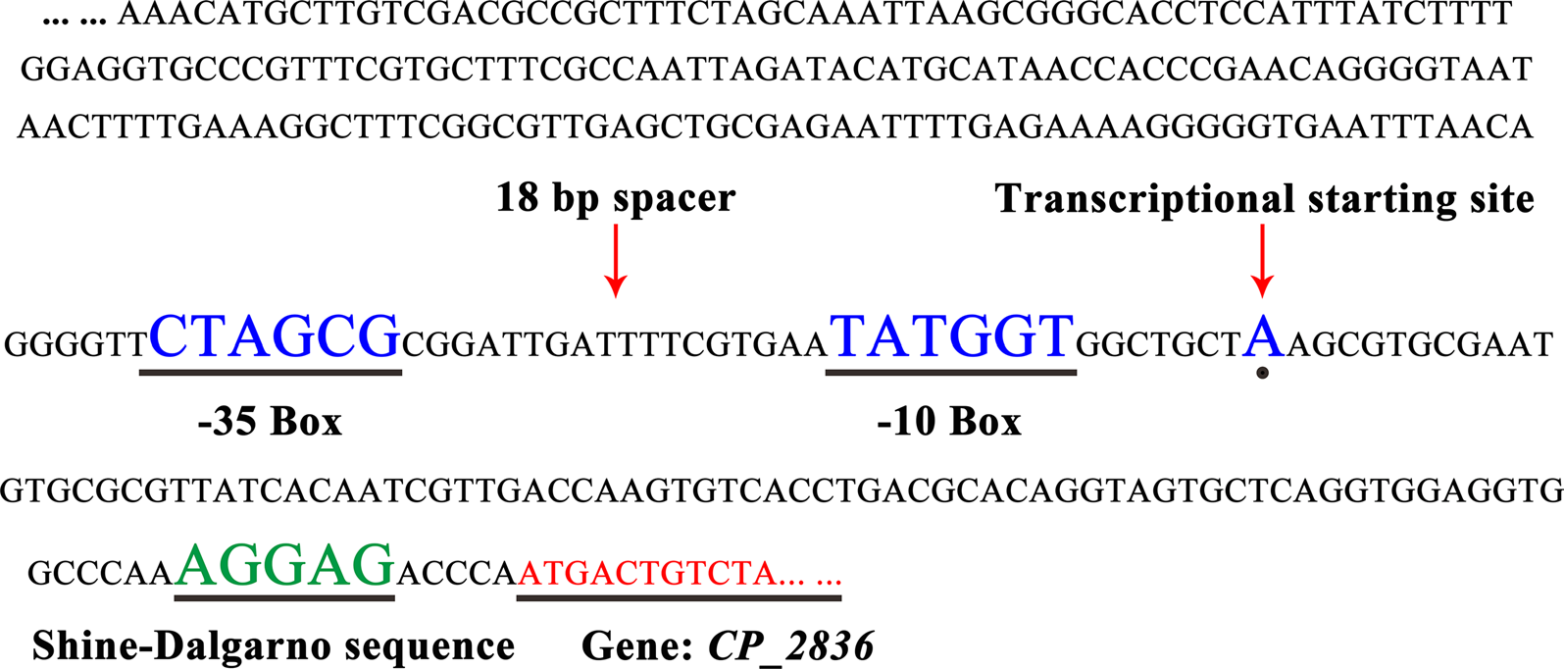
Sequence of the predicted P_CP_2836_ promoter. The − 35 box, − 10 box, spacer region, transcription start site, and SD-sequence were highlighted

**Fig. 2:**
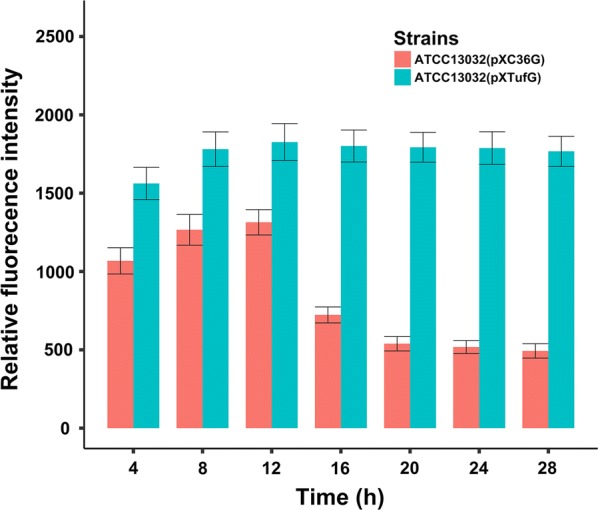
Expression of the green fluorescent protein (GFP) under control of the growth-regulated promoter or the *tuf* promoter by using plasmid. Cell fluorescence intensity was normalized to cell density. Measurement of unit fluorescence intensity of cells at 510 nm was performed using an ultraviolet–visible spectrophotometer. Mean ± standard deviations were calculated from three biological replicates

In order to assess the growth-regulated promoter P_CP_2836_, the green fluorescent protein (GFP) was used as a reporter to study the transcriptional strength of P_CP_2836_ throughout the entire culture process. Using the expression vector pXMJ19 as a backbone, the predicted sequences of P_CP_2836_ (Fig. 1) were cloned and ligated into pXMJ19 to replace the P_tac_ promoter of this vector. The GFP reporter gene was expressed under the control of P_CP_2836_ in reference to the *tuf* promoter in the wild-type *C. glutamicum* ATCC 13032. The strains were cultured for 28 h, where the cell densities and cell fluorescence intensities were measured every 4 h. The fluorescence intensity was normalized to cell density. As shown in Fig. 2, at 4, 8, and 12 h, the relative fluorescence intensity of ATCC 13032(pXC36-G) was about 75% that of ATCC 13032(pXTuf-G). As expected, however, the relative fluorescence intensity of ATCC 13032(pXC36-G) decreased at 16 h, and at 20–28 h it was only about 28% of ATCC 13032(pXTuf-G).

These results indicated that this promoter could be applied to regulate a target gene that needed to be highly expressed in the exponential growth phase but silent in the stationary growth phase (e.g. genes that are necessary for cell growth but are not related to target product synthesis). For the synthesis of l-valine, pyruvate is an important precursor which is also necessary for the TCA cycle. Under aerobic conditions, pyruvate entering the TCA cycle is mainly catalyzed by PDHC and citrate synthase [21]. The inactivation of PDHC and citrate synthase can cause an increase in l-valine production, however, it also has an irreversible negative impact on cell growth [15, 18]. Since most of the inducible promoters are tightly regulated with low basal expression before induction, it could not control the transcription of the *gltA* or *aceE* genes at a high level in the exponential phase and a low level in the stationary phase. In addition, there are rarely reports to inducible promoters which translation could be stopped or weakened after induction. Thus, by placing *aceE* or *gltA* under the control of this growth-regulated promoter, the TCA cycle should be active in the exponential growth phase (for cell growth) but be weakened in the stationary growth phase (increasing carbon availability).

### Application of the growth-regulated promoter to optimize the carbon metabolism of l-valine synthesis

**Fig. 3:**
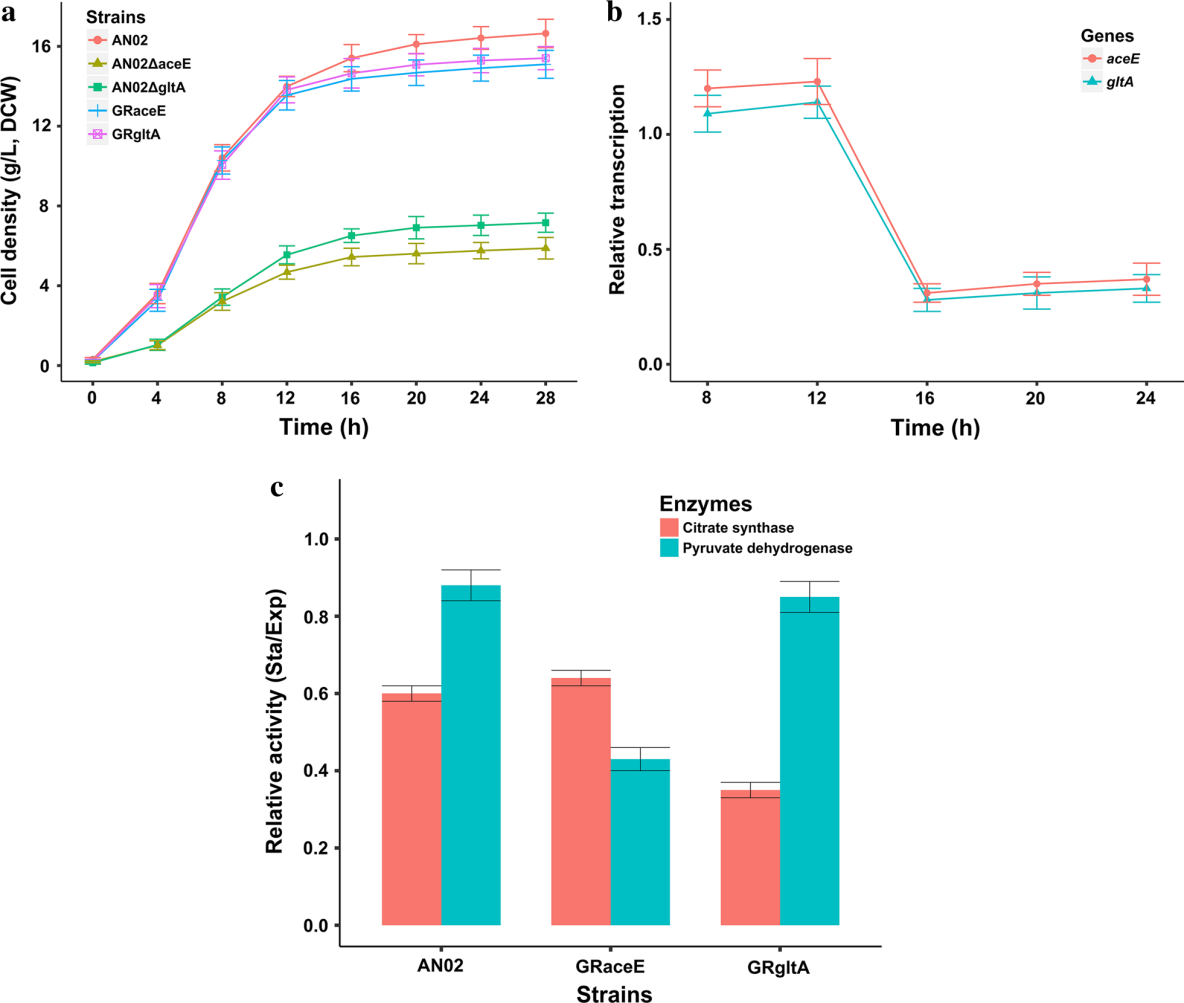
Application of the growth-regulated promoter to replace the native promoters of the *aceE* and *gltA* genes. **a** Cell densities of different strains. **b** Relative transcriptional levels of the *aceE* and *gltA* genes under the control of the growth-regulated promoter in reference to their native promoters. **c** Relative activity of pyruvate dehydrogenase and citrate synthase between stationary phase and exponential phase. *Exp* exponential phase, *Sta* stationary phase. Means ± standard deviations were calculated from three biological replicates

To verify this hypothesis, the promoter of the *aceE* or *gltA* gene of an l-valine producing strain, *C. glutamicum* AN02, was each replaced by the growth-regulated promoter P_CP_2836_ to generate the GRaceE and GRgltA strains, respectively. The l-valine producer AN02 was constructed in our previously study in which two copies of the feedback-inhibition-released acetolactate synthase coding gene were integrated into the genome of wild-type ATCC 13032 (unpublished data). Moreover, for reference, the *aceE* or *gltA* gene was also deleted from the genome of AN02 to construct a PDHC-deficient strain, AN02Δ*aceE*, and a citrate synthase-deficient strain, AN02Δ*gltA*, respectively. Strains were cultivated in flasks for 28 h. The final cell density, l-valine titer, glucose consumption, and the yield of substrate to product (Y_S/P_) are shown in Table 1. The final cell density of GRaceE and GRgltA was 7–10% lower than that of AN02. However, compared with AN02, the l-valine titer of GRaceE and GRgltA increased 23.9% and 27.3% and their Y_S/P_ increased 43.8% and 62.5%, respectively. As there was no extra acetate or citrate added into the culture, the cell densities and l-valine titers of AN02Δ*aceE* and AN02Δ*gltA* were both obviously lower than the other strains. However, GRaceE and GRgltA grew normally at 0–12 h, but showed a lower growth rate at 12–28 h compared with AN02 (Fig. 3a).

**Table 1:** The final cell density, l-valine production, glucose consumption and the yield of substrate to product of different strains after 28 h flask fermentation

Strains	Cell density (g/L)	l-Valine titer (g/L)	Glucose consumption (g/L)	Y_S/P_ (%)
AN2	16.65 ± 0.71	12.01 ± 0.36	112 ± 7	0.16
GRgltA	15.41 ± 0.58	15.29 ± 0.41	91 ± 6	0.26
GRaceE	15.10 ± 0.70	14.88 ± 0.33	99 ± 7	0.23
AN2Δ*gltA*	7.16 ± 0.48	3.3 ± 0.13	38 ± 5	–
AN2Δ*aceE*	5.88 ± 0.54	2.7 ± 0.16	34 ± 5	–

The transcriptional level of the *aceE* and *gltA* genes was assessed by fluorescence quantitative RT-PCR. At 8 and 12 h, the transcriptional level of the *aceE* gene of GRaceE was about 1.2-times higher than that of AN02; however, at 16–24 h, this ratio decreased to about 0.35 (Fig. 3b). Similarly, the expression ratio of the *gltA* gene of GRgltA to AN02 decreased from about 1.1 (8–12 h) to 0.30 (16–24 h). In addition, the activities of pyruvate dehydrogenase and citrate synthase decreased relative to their gene transcription. In GRaceE, the activity of pyruvate dehydrogenase, which was encoded by *aceE*, decreased in the stationary phase to about 43% of that of the exponential phase. While in GRgltA, the activity of citrate synthase, which was encoded by *gltA*, decreased to about 35% of that of the exponential phase (Fig. 3c).

The native promoters of the *aceE* and *gltA* genes are σ^A^-dependent promoters. The transcriptional pattern of this type of promoter decreases the transition between the exponential and stationary growth phases [2]. However, the change in the transcriptional strength of P_CP_2836_ was more sensitive to cell density. The transcriptional strength of P_CP_2836_ promoter was active throughout the exponential growth phase and rapidly decreased in the stationary growth phase. Moreover, the transcriptional difference of P_CP_2836_ between the exponential phase and the stationary phase was more apparent and its transcriptional strength was much lower in the stationary growth phase. Therefore, replacing the native promoters of the *aceE* and *gltA* genes with the P_CP_2836_ promoter contributed to optimize carbon availability of l-valine producers without any negative effect on cell growth.

### Effect of the growth-regulated promoter on carbon metabolism

**Fig. 4:**
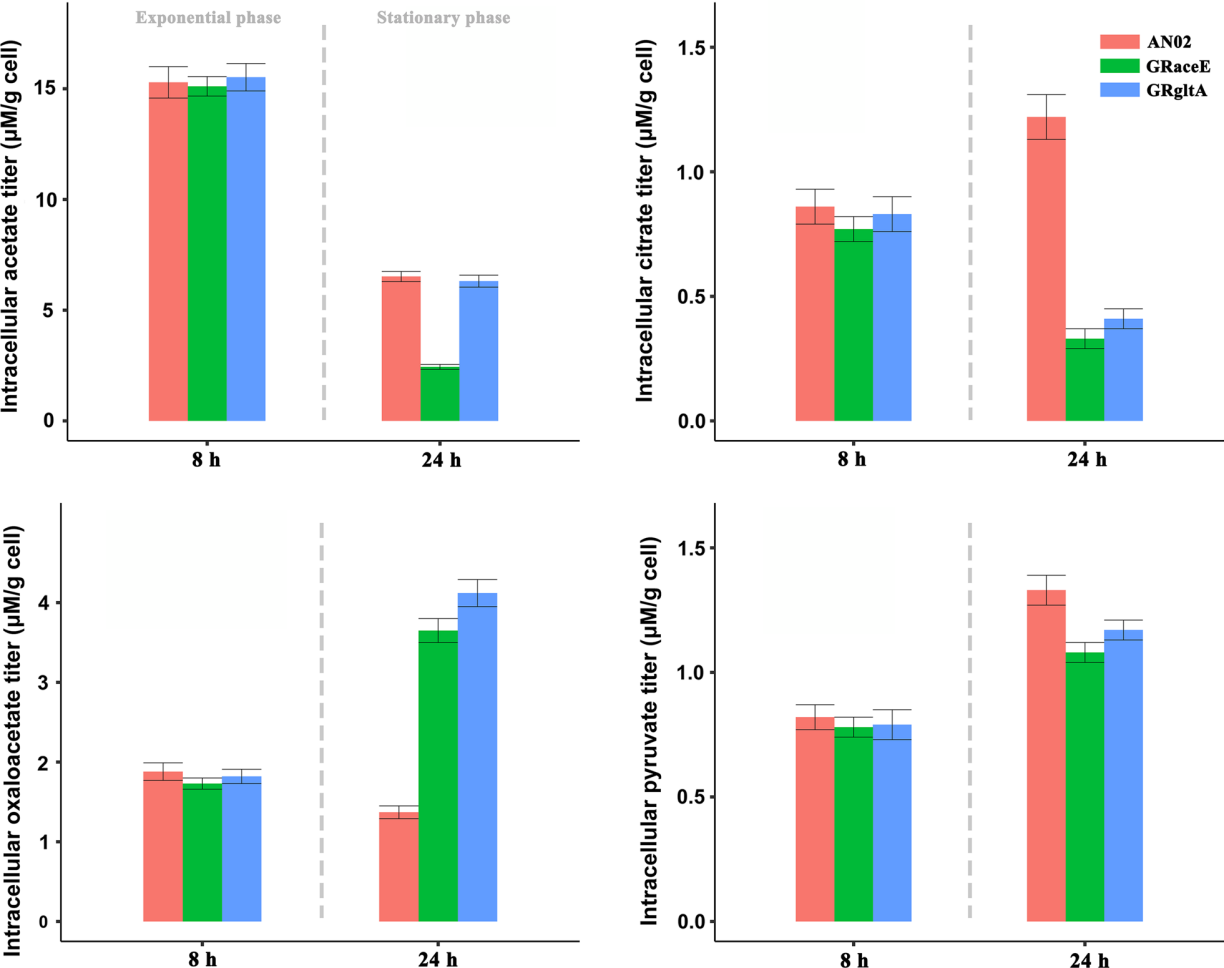
The intracellular levels (μM/g cell) of citrate, oxaloacetate, pyruvate, and acetate in the different growth phases. Data are presented as means ± standard deviation of three biological replicates

In central carbon metabolism pathway, PDHC and citrate synthase are two important nodes connecting the EMP pathway and the TCA cycle [22, 23]. To investigate the changes in the central carbon metabolism of GRaceE and GRgltA, we further measured the intracellular levels of pyruvate, citrate, oxaloacetate, and acetate. As shown in Fig. 4, the intracellular levels of these organic acids showed no obvious differences in the exponential phase (8 h). However, in the stationary phase (24 h), the intracellular citrate levels in GRaceE and GRgltA decreased to 27.0% and 33.6% of that of AN02, respectively. Meanwhile, the intracellular oxaloacetate levels in GRaceE and GRgltA increased to 2.7 and 3.0 times of that of AN02, respectively. These results indicated that the metabolism rate of the TCA cycle of GRaceE and GRgltA were lower than that of AN02 [24]. Moreover, the intracellular pyruvate levels in GRaceE and GRgltA were slightly decreased in the stationary phase compared with AN02. This was probably due to the impact of the decreased metabolism rate of the TCA cycle relative to central carbon metabolism. In addition, the intracellular acetate level of GRaceE was significantly lower than that of GRgltA and AN02 in the stationary phase. The synthesis of acetate depends on acetyl-CoA, which is formed from pyruvate and is catalyzed by PDHC [25]. As such, it was apparent that the promoter replacement caused the activity of PDHC of GRaceE to decrease to the point that sufficient acetyl-CoA could not be provided for acetate synthesis. These results indicated that the activities of the PDHC and citrate synthase decreased in the stationary growth phase and corresponded to the change in transcriptional strength of the growth-regulated promoter. Thus, limited pyruvate entered into the TCA cycle. On the other hand, more pyruvate was available to be used for synthesis of the target product. Therefore, both production and yield of l-valine increased.

Our promoter replacement strategy overcame the serious, irreversible, and negative impact on cell growth that would occur following a knockout of the *aceE* or *gltA* genes. In addition, as this promoter would not require an inducer or any change in the fermentation conditions, it served as an authentic auto-regulation system. This strategy also served to reduce the workload and the risk of contamination and helped ensure the stability of the batch fermentation [26, 27]. Furthermore, this growth-regulated promoter could be used in a number of different metabolic engineering projects to control gene expressing by conferring different transcriptional levels to the different growth phases. Importantly, the development of such growth-regulated promoters can enhance the selectivity and timing of gene expression for use in metabolic engineering, and these could serve as important tools for use in many aspects of basic research.

## Conclusions

This study identified the *C. glutamicum* growth-regulated promoter P_CP_2836_, which demonstrated a significant difference in transcriptional strength between the exponential and stationary growth phases. The transcriptional strength of P_CP_2836_ was about 75% that of the strong constitutive promoter, P_tuf_, in the exponential growth phase, but only about 8% of P_tuf_ in the stationary growth phase. By replacing the native promoters of the *aceE* and *gltA* genes with P_CP_2836_ in the l-valine-producing strain *C. glutamicum* AN02, the transcriptional levels of these genes and the activity of their translational products were both significantly decrease in the stationary growth phase. In addition, in the modified strains GRaceE and GRgltA, the l-valine titers increased by 23.9% and 27.3% and the yield of substrate to product increased 43.8% and 62.5%, respectively. Intracellular organic acid analysis indicated that the metabolism rate of the TCA cycle in the engineered strains decreased. Additionally, compared with a direct knockout of the *aceE* or *gltA* gene, the engineered strains did not demonstrate any serious or irreversible negative impacts on cell growth. Besides, to place both the *aceE* and *gltA* genes under the control of the P_CP_2836_ promoter is expected to further increase the l-valine production yield, which will be used to maximize the production of l-valine. The identification of this growth-regulated promoter serves to enhance the selectivity and timing of gene expression in *C. glutamicum*.

## Materials and methods

### Strains and plasmids

The strains and plasmids used in this study are listed in Table 2. *C. glutamicum* CP is an industrial l-leucine-producing strain obtained by repeated mutagenesis and selection. This strain is accessible at the China General Microbiological Culture Collection Center with the identifier CGMCC 11425. The wild-type *C. glutamicum* ATCC 13032 and the plasmids pXMJ19, pK18mobsacB, and pCmGFP are stored in our laboratory.

**Table 2:** Bacterial strains and plasmids used in this study

Strains and plasmids	Relevant characteristics^a^	Source
*C. glutamicum* strains		
CP	l-Leucine-producing, Ile^−^, 2-TA^r^, SG^r^, LeuHx^r^, NV^r^	Lab store
ATCC13032	Wild-type strain	Lab store
ATCC 13032(pXTuf-G)	*C. glutamicum* ATCC13032 harbouring pXTuf-G, Cm^r^	This study
ATCC 13032(pXC36-G)	*C. glutamicum* ATCC13032 harbouring pXC36-G, Cm^r^	This study
AN02	*C. glutamicum* ATCC13032, Δ*Cgl2610*::P_tuf_+*ilvBN*^XV^, Δ*Cgl1890*::P_tuf_+*ilvBN*^XV^	Lab store
AN02Δ*aceE*	*C. glutamicum* AN02 Δ*aceE*	This study
AN02Δ*gltA*	*C. glutamicum* AN02 Δ*gltA*	This study
GRaceE	*C. glutamicum* AN02 ΔP_aceE_:: P_CP_2836_	This study
GRgltA	*C. glutamicum* AN02 ΔP_gltA_:: P_CP_2836_	This study
Plasmids		
pXMJ19	Cm^r^; P_Tac_ promoter	[30]
pK18mobsacB	Km^r^; *sacB*	[28]
pCmGFP	Carrying the sequence of green fluorescent protein (GFP)	Lab store
pXTuf-G	Cm^r^; pXMJ19 ΔP_Tac_::(P_tuf_+GFP)	This study
pXC36-G	Cm^r^; pXMJ19 ΔP_Tac_::(P_CP_2836_+GFP)	This study
pK18-aceE	Km^r^; pK18mobsacB derivative containing the upstream and downstream regions of gene *aceE*	This study
pK18-gltA	Km^r^; pK18mobsacB derivative containing the upstream and downstream regions of gene *gltA*	This study
pK18-GRaceE	Km^r^; pK18mobsacB derivative containing P_CP_2836_ promoter covered by upstream and downstream regions of the promoter region of *aceE*	This study
pK18-GRgltA	Km^r^; pK18mobsacB derivative containing P_CP_2836_ promoter covered by upstream and downstream regions of the promoter region of *gltA*	This study

^a^Ile: l-isoleucine; LeuHx: l-leucine hydroxamate; NV: norvaline; SG: sulfaguanidine; 2-TA: 2-thiazolylalanine; Cm: chloromycetin; Km: kanamycin

### Nucleotide sequence accession numbers

The genome of *C. glutamicum* CP is accessible at GenBank with the accession number CP012194.1. The RNA-seq data of *C. glutamicum* CP have been deposited in the NCBI Sequence Read Archive (SRA) under accession number SRP143929.

### Construction of plasmids and strains

The primers used in this study are listed in Additional file 1. The PCR was performed using the PrimeSTAR^®^ HS DNA Polymerase (TaKaRa, Dalian, China). The parameter settings for amplification of DNA were 94 °C for 5 min, 30 cycles of 98 °C for 10 s, 55 °C for 5 s, and 72 °C for 1 min/Kb, and finally 72 °C for 10 min. General DNA manipulations were performed using the Takara QuickCut Enzyme (TaKaRa) for linearization of plasmid DNA and the ClonExpress II One Step Cloning Kit (Vazyme Biotech, Nanjing, China) for DNA ligation. To construct pXTuf-G, the pXMJ19 plasmid was linearized via digestion with the *Hpa*I and *Kpn*I restriction enzymes, where the P_tac_ promoter was removed. The sequence of the P_tuf_ promoter was amplified by PCR from the *C. glutamicum* CP genome using primers 1/2, and the green fluorescent protein (GFP) coding gene *gfp* was amplified by PCR from the pCmGFP plasmid using primers 3/4. Then, the PCR products were ligated by overlapping PCR using primers 1/4 and further ligated into the linearized pXMJ19 plasmid. The pXC36-G plasmid was constructed with a similar method using primers 3–6. These plasmids were then transformed into *C. glutamicum* ATCC 13032 to overexpress GFP.

Chromosomal DNA manipulations were achieved via a markerless system using the suicide vector pK18mobsacB, as previously described [28]. To construct pK18-GRaceE, the pK18mobsacB plasmid was linearized via digestion with the *Hin*dIII and *Eco*RI restriction enzymes. The sequence of the P_CP_2836_ promoter was amplified by PCR from the *C. glutamicum* CP genome using primers 7/8, and the up- and down-stream sequences of the *aceE* gene promoter were amplified by PCR from the *C. glutamicum* ATCC 13032 genome using primers 9/10 and 11/12. Then, the PCR products were ligated by overlapping PCR using primers 9/12 and ligated into the linearized pK18mobsacB plasmid. The pK18-GRaceE plasmid was transformed into *C. glutamicum* AN02 by electroporation, and the native promoter of *aceE* was replaced by P_CP_2836_ via homologous recombination in the form of a double crossover to obtain the GRaceE strain. The same method was used to replace the *gltA* promoter with P_CP_2836_ to obtain the GRgltA strain and to knockout the *aceE* and *gltA* genes to obtain the AN02Δ*aceE and* AN02Δ*gltA* strains. All DNA manipulations, including chromosomal editing and plasmid construction, performed in this work were verified by Sanger sequencing to ensure no extra DNA changes were introduced.

### Fermentation and analytical procedures

Bacteria were cultivated in 500-mL flasks at 32 °C in medium containing 8% (w/v) glucose, 0.5% (w/v) yeast extract, 1% (w/v) (NH_4_)_2_SO_4_, 0.2% (w/v) K_2_HPO_4_, 0.2% (w/v) KH_2_PO_4_, 0.1% (w/v) MgSO_4_, 0.001% (w/v) FeSO_4_, 0.001% (w/v) MnSO_4_, 0.001% (w/v) biotin, and 0.001% (w/v) thiamine for 28 h to produce l-valine. During the fermentation process, the pH was maintained at approximately 7.0 using NH_4_OH (25%, v/v) and the concentration of glucose in the culture was maintained at not less than 2% (w/v) by feeding with glucose (80%, w/v).

Amino acid contents were measured by high performance liquid chromatography using an Agilent ZORBAX Eclipse AA column (4.6 mm × 150 mm, 5-µm; Agilent Technologies, Palo Alto, CA, USA) with detection at 360 nm. Acetate buffered acetonitrile was used as the mobile phase at a flow rate of 1 mL/min.

### RNA extraction and sequencing

Total RNA was extracted (three independent repeats per condition) using the Eastep Super Total RNA Extraction Kit (Promega, Shanghai, China) according to the manufacturer’s instructions. RNA concentrations were determined by Qubit fluorometer (ThermoFisher Scientific, Waltham, USA). Sequencing libraries were generated using NEBNext^®^ Ultra™ RNA Library Prep Kit for Illumina^®^ (NEB, Ipswich, USA) following the manufacturer’s recommendations, and index codes were added to attribute sequences to each sample. The library preparations were sequenced by Illumina HiSeq 2500 platform (Illumina, San Diego, USA) and 100 bp paired-end reads were generated. After data filtering and quality assessment, there were more than 7 million clean reads and more than 1 Gb clean bases for each sample.

### Preparation of cDNA and fluorescence quantitative reverse transcription-polymerase chain reaction (RT-PCR)

Total RNA was extracted from collected cells using the Eastep Super Total RNA Extraction Kit (Promega, Shanghai, China), according to the manufacturer’s instructions. Then, total RNA was reversely transcribed to cDNA using the PrimeScript RT Master Mix (TaKaRa, Dalian, China). The transcriptional levels of different genes were identified by fluorescence quantitative RT-PCR using the cDNA as the template. The fluorescence quantitative RT-PCR was performed using the SYBR^®^ Premix Ex Taq™ II (Tli RNaseH Plus; TaKaRa, Dalian, China). The primers for RT-PCR are listed in Additional file 1. The parameter settings for amplification of DNA were as follows: 95 °C for 30 s, 40 cycles of 95 °C for 5 s, and 60 °C for 35 s, and for the dissociation stage were 95 °C for 15 s, 65 °C for 35 s, and 95 °C for 15 s. The transcriptional level of 16S ribosomal RNA was used as an internal reference.

### Measurement of cell fluorescence intensity

Cell culture was diluted to an OD_600_ value of 0.2–0.6 to measure cell fluorescence intensity. A FL F-7000 ultraviolet–visible spectrophotometer (Hitachi, Beijing, China) was used to detect cell fluorescence at an excitation wavelength of 510 nm.

### Assay of enzyme activities

For enzyme activity assays, cells were collected by centrifugation (10,000×*g*, 5 min, 4 °C), and each 100 mg wet cell pellet was re-suspended in 1 mL ice-cold Tris/HCl buffer (50 mM, pH 7.5). Then, 100 μL lysozyme (50 mg/mL in distilled water) was added into a 4 mL cell suspension, and the cell suspensions were incubated at 32 °C for 15 min. The protoplasts were collected by centrifugation (10,000×*g*, 5 min, 4 °C) and washed twice with Tris/HCl buffer. The protoplasts were then re-suspended to 100 mg wet cells/mL in ice-cold Tris/HCl buffer and subjected to ultrasonic homogenization in an ice bath. The homogenates were centrifuged (15,000×*g*, 15 min, 4 °C), and the supernatants were used for enzyme activity assays.

Pyruvate dehydrogenase was assayed using the Pyruvate Dehydrogenase (PDH) Enzyme Activity Microplate Assay Kit (Abcam, Shanghai, China), according to the manufacturer’s instructions. Citrate synthase was assayed by the method described in Lemos et al. [29]. First, 520 μL of buffer (containing 50 mM Tris/HCl with 100 mM KCl and 1 mM EDTA, pH 7.5), 20 μL DTNB (5,5′-dithio-bis-2-nitrobenzoic acid, 5.025 mM in sample buffer), 20 μL acetyl coenzyme A (2.5 mM in distilled water), and 20 μL of the crude enzyme were mixed in a 1.5 mL EP tube. After 5 min of preincubation at 25 °C, the reaction was initiated with the addition of 20 μL oxaloacetate (5.0 mM in distilled water), and the increase in absorbance at 412 nm was registered.

### Intracellular organic acid analysis

Samples for metabolic analysis were collected at the exponential growth (at 8 h) and stationary growth (at 24 h) phases. Cells were centrifuged (10,000×*g*, 5 min, 4 °C) and washed with 50 mL 0.9% NaCl at 4 °C. After centrifugation (10,000×*g*, 5 min, 4 °C), 50 mg wet cell pellets were re-suspended in 1 mL PBS buffer (pH 8.0, 4 °C), and 20 μL deuterium-labeled succinate (1 mg/mL) was added as an internal standard. Intracellular metabolites were extracted via five freeze–thaw cycles and ultrasonication (10 min, 4 °C). Finally, the solvent mixture was centrifuged (10,000×*g*, 5 min, 4 °C) and 1 mL of the supernatant was transferred to a new 1.5-mL tube and completely dried in a Vacuum Freeze Dryer (Gold-Sim, Los Angeles, USA). The dried samples were derivatized with 40 mg/L methoxyamine in 20 mL pyridine at 30 °C for 90 min and 45 µL of *N*-methyl-*N*-(trimethylsilyl)trifluoroacetamide (MSTFA) (Sigma-Aldrich, St. Louis, USA) at 37 °C for 30 min. An Agilent 7890A-5975C GC–MS system (Agilent Technologies, Santa Clara, USA) and an HP-5 ms column (60 m × 0.32 mm, 0.25-μm film thickness; Agilent Technologies, Santa Clara, USA) were used for metabolite analysis. The sample was injected into the GC under split mode (helium total flow rate 9 mL/min with a sweeping rate of 3 mL/min). The initial oven temperature was 70 °C for 2 min and then increased to 290 °C for 4 °C/min. Mass spectra were recorded using full scan mode. The temperatures of the injection port, ion source, transfer line, and quadrupoles were set at 250 °C, 230 °C, 280 °C, and 150 °C, respectively.

## Additional file

**Additional file 1.** Primers used in this study.
