# Supplementary material for: Identification and application of a growth-regulated promoter for improving l-valine production in Corynebacterium glutamicum
Source: Microb Cell Fact. 2018 Nov 24;17:185. doi: 10.1186/s12934-018-1031-7 (PMC6260661; doi:10.1186/s12934-018-1031-7)
Supplement: Supplementary file 1 — Additional file 1. Primers used in this study. [file 12934_2018_1031_MOESM1_ESM.pdf]

Identification and application of a growth-regulated promoter in *Corynebacterium glutamicum*

Yuechao Ma[1,2], Yi Cui[1,2], Lihong Du[1,2], Xiaoqian Liu[1,2], Bo Yang[3], Xixian Xie[1,2*], Ning Chen[1,2*]

1. National and Local United Engineering Lab of Metabolic Control Fermentation Technology, Tianjin University of Science & Technology, Tianjin 300457, PR China

2. College of Biotechnology, Tianjin University of Science & Technology, Tianjin 300457, PR China

3. The Institute of Seawater Desalination and Multipurpose Utilization, SOA Tianjin, Tianjin 300192, PR China

***Corresponding author:**

Dr. Xixian Xie, College of Biotechnology, Tianjin University of Science & Technology, No. 29, 13 Main Street, Tianjin Economic and Technological Development Area, Tianjin 300457, PR China; Tel: 86-22-60601251; Fax: 86-22-60602298; E-mail: xixianxie@tust.edu.cn

Dr. Ning Chen, College of Biotechnology, Tianjin University of Science & Technology, No. 29, 13 Main Street, Tianjin Economic and Technological Development Area, Tianjin 300457, PR China; Tel: 86-22-60601251; Fax: 86-22-60602298; E-mail: ningch@tust.edu.cn

Email addresses of other authors:
Yuechao Ma: maychao123@163.com
Yi Cui: cy18303095435@163.com
Lihong Du: lihongdu1010@163.com
Xiaoqian Liu: lxq13043122@126.com
Bo Yang: bobo709@yeah.net

Additional file 1. Primers used in this study.

Primer no.	Target gene	Sequence (5'-3')
1	*tuf* promoter	ATGTTATATCCCGCCGTTAACAGATCGTTTAGATCCGAAGGAAAA
2		GAAAAGTTCTTCTCCTTTACTCATTCGTAGCCACCACGAAGTCC
3	GFP	ATGAGTAAAGGAGAAGAACTTTTC
4		TCTAGAGGATCCCCGGGTACCTTATTTGTATAGTTCATCCATGCCATG
5	P_{CP_2836} promoter	ATGTTATATCCCGCCGTTAACAAAACATGCTTGTCGAC
6		GAAAAGTTCTTCTCCTTTACTCATTGGGTCTCCTTTGGG
7	P_{CP_2836} promoter	CCGATTGAAAACATGCTTGTCGAC
8		TCGGCCATTGGGTCTCCTTTGGGC
9	*aceE* promoter Upstream	TATGACCATGATTACGAATTCCGATTACATGGATTCAAC
10		CATGTTTTCAATCGGTGTGCTTC
11	*aceE* promoter Downstream	GAGACCCAATGGCCGATCAAGCAA
12		ACGACGGCCAGTGCCAAGCTTGGCTGCGCCTGCGTAAG
13	P_{CP_2836} promoter	TGTGCACCAAACATGCTTGTCGAC
14		CAAACATTGGGTCTCCTTTGGG
15	*gltA* promoter Upstream	TATGACCATGATTACGAATTCGAAGGTCCGCAGCCG
16		GCATGTTTGGTGCACAGTTTAGC
17	*gltA* promoter Downstream	AGACCCAATGTTTGAAAGGGATAT
18		ACGACGGCCAGTGCCAAGCTTATTGGGTGAGCGTCGCG
19	*aceE* Upstream	CTATGACATGATTACGAATTCCGACCAAGAATGGGACCG
20		TGTTGGCAAAGCCAACTTCGTACAGAGGG
21	*aceE* Downstream	GTTGGCTTTGCCAACATTTACTCCGCTACT
22		ACGACGGCCAGTGCCAAGCTTTTTGGATCTACGGAAACACTCG
23	*gltA* Upstream	CTATGACATGATTACGAATTCTGAATGCTGGCCTTGAGGG
24		TGCGGGATGGTCAAAAGTGATCAGTCCAGTCTC
25	*gltA* Downstream	CTTTTGACCATCCCGCAAGTTGGTTCCTC
26		ACGACGGCCAGTGCCAAGCTTAGACAGTGGGTGGCCGGA
RT-1	*tuf*	CAGGACTACGACGAAGAGG
RT-2		ACGAACTGGGTCAGGGAT
RT-3	*CP_2836*	CCACTTCCGCTACTTTGC
RT-4		CTGACCAACAACACCGATT
RT-5	*aceE*	CTTCGGTCTTGACTCTTGGTT
RT-6		AACGATGCCACGGAACCAG
RT-7	*gltA*	ATGCGGGAATCCTGCGTTAC
RT-8		GTTGCCATTGGGTGAGCGTC
RT-9	16S ribosomal RNA	CGATACGGGCATAACTTGA
RT-10		GTTTACGGCATGGACTACC
